# Face puzzle—two new video-based tasks for measuring explicit and implicit aspects of facial emotion recognition

**DOI:** 10.3389/fpsyg.2013.00376

**Published:** 2013-06-26

**Authors:** Dorit Kliemann, Gabriela Rosenblau, Sven Bölte, Hauke R. Heekeren, Isabel Dziobek

**Affiliations:** ^1^Cluster of Excellence “Languages of Emotion”, Freie Universität BerlinBerlin, Germany; ^2^Department of Education and Psychology, Freie Universität BerlinBerlin, Germany; ^3^Center of Neurodevelopmental Disorders (KIND), Department of Women's and Children's Health, Karolinska InstitutetStockholm, Sweden; ^4^Division of Child and Adolescent Psychiatry, Stockholm County CouncilStockholm, Sweden

**Keywords:** social cognition, implicit, explicit, faces, emotion recognition, autism spectrum disorder

## Abstract

Recognizing others' emotional states is crucial for effective social interaction. While most facial emotion recognition tasks use explicit prompts that trigger consciously controlled processing, emotional faces are almost exclusively processed implicitly in real life. Recent attempts in social cognition suggest a dual process perspective, whereby explicit and implicit processes largely operate independently. However, due to differences in methodology the direct comparison of implicit and explicit social cognition has remained a challenge. Here, we introduce a new tool to comparably measure implicit and explicit processing aspects comprising basic and complex emotions in facial expressions. We developed two video-based tasks with similar answer formats to assess performance in respective facial emotion recognition processes: *Face Puzzle, implicit and explicit*. To assess the tasks' sensitivity to atypical social cognition and to infer interrelationship patterns between explicit and implicit processes in typical and atypical development, we included healthy adults (NT, *n* = 24) and adults with autism spectrum disorder (ASD, *n* = 24). Item analyses yielded good reliability of the new tasks. Group-specific results indicated sensitivity to subtle social impairments in high-functioning ASD. Correlation analyses with established implicit and explicit socio-cognitive measures were further in favor of the tasks' external validity. Between group comparisons provide first hints of differential relations between implicit and explicit aspects of facial emotion recognition processes in healthy compared to ASD participants. In addition, an increased magnitude of between group differences in the implicit task was found for a speed-accuracy composite measure. The new Face Puzzle tool thus provides two new tasks to separately assess explicit and implicit social functioning, for instance, to measure subtle impairments as well as potential improvements due to social cognitive interventions.

## Introduction

To effectively function as social agents, humans must process information in the social environment in order to initiate immediate behavioral responses. One important source of information concerning the internal states of others is provided in emotional facial expressions. Complex social information is not always obviously present, nor are we usually confronted with explicit prompts to interpret the information (“do you think the smile is real?”). Instead, a large fraction of subtle social information must be automatically recognized and integrated, such as the fine differences between a genuine smile (“Duchenne,” involving the contraction of both the zygomatic major muscle, which raises the corner of the mouth, *and* the orbicularis oculi muscle, which raises the cheeks) and a fake smile (“non-Duchenne,” only contracting the zygomatic major muscle) (Ekman et al., 1990; Frank et al., 1993). To successfully read the emotions of others, we need to both implicitly and explicitly process aspects of the social world.

A formal theoretical dissociation of implicit and explicit processes has been proposed in the context of general knowledge within cognition, whereby implicit knowledge is assumed to be a precursor to explicit knowledge in development (Dienes and Perner, 1999; Perner and Dienes, 2003), by the re-description of implicit representations to explicit knowledge (Karmiloff-Smith, 1992). These approaches have recently been translated into attempts to dissociate explicit and implicit processes in the field of social cognition, such as a dual-process model of mental state inferences, i.e., Theory of Mind (ToM), which postulates an earlier developing implicit and a later developing explicit ToM system (Apperly and Butterfill, 2009; Low and Perner, 2012). The question remains whether and to what extent respective postulated distinctions within higher-level social cognitive constructs (e.g., ToM) can be applied to the processing of more basic types of social stimuli, such as emotional faces.

Social cognition (i.e., cognitive mechanisms that underlie social behavior) in its explicit form is usually concerned with conscious and controlled processing, which is rather flexible, but also demands many cognitive resources. In contrast, implicit social cognition is usually considered to be more automatic and time efficient, but inflexible and limited in terms of cognitive resources. The well-known and often applied definition of *implicit* according to Greenwald and Banaji (1995) (within the field of social psychology) implies that implicit processes occur outside of conscious awareness. There have been, however, re-considerations of this definition of implicit as purely unconscious because unawareness of the tested psychological construct is not always guaranteed or considered to be a necessary criterion (Fazio and Olson, 2003; Nosek et al., 2011, 2012). Instead, it has been proposed that measures approximating implicit socio-cognitive processes share a more indirect assessment without directly asking individuals for a verbal report (Fazio and Olson, 2003).

Applying this distinction of implicit and explicit processes, standard emotion recognition tasks are approximating explicit emotion recognition processes. Usually some type of visual facial stimulus (such as pictures of faces or parts of faces) is presented, and participants then have to consciously process and choose between emotional words in order to label the expression in a controlled fashion. Thus, participants have to match a target with a label by explicitly comparing the emotional aspects of the facial expression with the provided linguistic/verbal concepts of emotional expressions. One advantage of these tasks is that they provide a direct performance-based measure to depict behavior or related impairments in real life. In everyday interactions, however, emotions must be recognized without the explicit comparison with provided emotional labels that involve specific emotional concepts.

In contrast, implicit processes during facial emotion perception are usually assessed indirectly. Thereby, the effect of an emotional expression on another psychological construct, e.g., racial attitudes, gender judgments, or attractiveness ratings (see, e.g., Devine et al., 2002; Amodio et al., 2004) are most often assessed indirectly via reaction times or eye movement patterns in response to the stimuli. Other indirect measures, for instance, investigate the influence of particular social cues (e.g., facial expressions or gaze direction) on judgments about body orientation or positioning (Hudson et al., 2009, 2012; Hudson and Jellema, 2011). However, when investigating subtle interindividual differences or impairments in a psychological construct, accuracy scores are of great value (Zaki and Ochsner, 2011). In particular, methodological comparability of task formats when comparing individual performance approximating implicit vs. explicit processes is indispensable, yet remains a challenge.

Here, we assessed the implicit and explicit processing of facial emotion recognition directly with comparable performance measures. The newly developed Face Puzzle explicit task triggers emotion recognition from facial expressions explicitly by instructing participants to match videos of emotional faces with verbal labels. The explicit task thus comprises controlled and conscious comparisons of facial emotion stimuli and provided emotional concepts in terms of verbal labels. In contrast, there were no such explicit prompts to identify a specific facial emotional expression in the implicit Face Puzzle task. Instead, in the implicit task participants must identify emotional cues in parts of the face to correctly compose a complete facial expression from puzzle pieces more indirectly without being provided with possible verbal labels or asking for a verbal report of the emotional concept.

We are aware of the fact that our approach of puzzling faces without explicit verbal prompts and direct consideration of multiple answering options reduces the concept of ‘implicit’ to a very specific and somewhat narrow conceptualization (see, e.g., Moors et al., 2010). Nevertheless, in order to advance our understanding of facial emotion recognition by comparing and quantifying implicit and explicit aspects of emotion processing, we deem this approach to be warranted.

To inform the sensitivity of new social cognition tasks, the study of disorders involving selective socio-affective impairments, such as high-functioning autism spectrum disorder (ASD), can be valuable. ASD is a neurodevelopmental condition that comprises deficits in social communication, social interaction, and repetitive behaviors (Levy et al., 2009). While many studies report deficits in explicit emotion labeling in ASD, non-performance-based measures strongly indicate that ASD involves particularly highlighted impairments in implicit aspects of social cognition involving mental or emotional state inferences (e.g., Kliemann et al., 2010; Yoshida et al., 2010; Kirchner et al., 2012; Senju, 2012) or non-mental state inferences [e.g., reduced imitation, facial mimicry, interpretation of social cues, (McIntosh et al., 2006; Jellema et al., 2009; Senju, 2013)]. Despite these reports of greater impairments in implicit as compared to explicit socio-cognitive functions in ASD, the results remain inconclusive, and direct evidence for this hypothesis via a comparison with performance in explicit processes is lacking. This gap further suggests a need to develop new tests that offer an empirical foundation to attempt a dissociation of implicit and explicit aspects of facial emotion recognition in typical and atypical samples. New performance-based measures that assess implicit processing could also inform the heterogeneity in the social phenotype of ASD and other affective disorders due to individual differences in the interactions of implicit and explicit impairments.

The aim of the current study was to develop a new tool to comparably measure implicit and explicit aspects of behavioral emotional face processing abilities: Face Puzzle, implicit and explicit. In order to (1) test the new tasks' sensitivity to atypical social cognition and (2) inform possible dissociations between implicit and explicit processes, we included a high-functioning ASD sample and used more naturalistic video stimuli, as opposed to pictures of still faces or face parts. With respect to previous studies' results of greater deficits in implicit as compared to explicit social processing in ASD, we expected the performance of individuals with ASD to differ to a greater extent from controls in the implicit task as compared to the explicit task.

## Materials and methods

### Participants

Twenty-four neurotypically developed participants (15 male, mean age = 30.3 years, *SD* = 8.37) with no history of psychiatric or neurological disorders and 24 adults on the autism spectrum (15 male, mean age = 30.4 years, *SD* = 8.52) participated in the current study. Control participants were recruited by public notices and from project databases of the Freie Universität Berlin, Germany. ASD participants were recruited through the autism in adulthood outpatient clinic of the Charité University Medicine Berlin, Germany or were referred to us by specialized clinicians. Diagnoses were made according to DSM-IV criteria for Asperger disorder and autistic disorder without intellectual disabilities using two instruments known to be the gold standard for diagnosing autism: the Autism Diagnostic Observation Schedule (ADOS, Lord et al., 2000) and the Autism Diagnostic Interview-Revised (ADI-R, Lord et al., 1994) if parental informants were available. For 21 individuals, the diagnosis Asperger syndrome was additionally confirmed with the Asperger disorder and High Functioning Autism Diagnostic Interview (ASDI, Gillberg et al., 2001).

In addition to age and gender, groups were matched with respect to their intelligence level. Age specific approximations of fluid and crystalline intelligence were assessed with a German vocabulary test [Mehrfach-Wortschatz-Test (MWT), (Lehrl, 1995)] and a strategic thinking test (LPS, subscale 4, Horn, 1962), respectively. To control for clinically significant levels of autistic traits in healthy populations, we applied the Autism Spectrum Quotient (AQ, Baron-Cohen et al., 2001b; German translation: Freitag et al., 2007) in both groups. All participants had normal or corrected-to-normal vision and were native German speakers. Participants gave written informed consent prior to participation and received payment for their time. The study was approved by the ethics committee of the German Society for Psychology (DGPs).

### Stimulus production and validation

Stimulus production was part of a comprehensive project to produce a new set of more ecologically valid video stimulus material, comprising a total set of 40 different emotional states that were depicted in facial expressions by more than 50 professional actors of varying age (18–65 years) at the film studio of the Humboldt University, Berlin, Germany, in cooperation with its Computer and Media Service (CMS). Selection of the 40 emotions was based on a previous study that characterized emotional words not only based on the classic valence and arousal dimensions but also regarding their frequency and thus relevance in everyday life (communicative frequency, see, Hepach et al., 2011).

Video clips show the actor oriented toward the camera making an emotional expression with a neutral on- and offset. The final stimulus set comprises 1910 videos, approximately 45 per emotional state. The actors were given specific emotion inducing instructions, comprising, e.g., situations in which the respective emotion usually occurs (e.g., expectant: “your fiancé comes back from a long business trip and you can't wait to see him/her again”) and physiological information (i.e., “you are so excited that your heartbeat increases and your hands get sweaty”). Actors were further invited to remember a personal event and to imaginatively put themselves in that event, including allowing original emotional reactions to arise. Actor instructions were developed in close interaction with professional acting instructors.

Stimuli were subjected to several validation steps: quality of expression (e.g., believability and preciseness) was evaluated first during stimuli production and second during cutting of recorded material. If videos were identified as insufficient/invalid they were immediately excluded from the dataset. To select stimuli for the Face Puzzle tasks from the complete video set, we selected videos from 20 actors spanning various ages and randomly selected 100 videos (20 actors, 10 male, 5 emotions, 2 positive) to test their validity in a separate expert validation study (10 psychologists working in the field of social cognition, 4 male, mean age = 29.6 years, *SD* = 4.3). The results showed high average emotion recognition rates (92.6%, *SD* = 0.07) and good believability [mean = 4.4, *SD* = 0.07; 6-point Likert scale (1 = not believable to 6 = very believable)] of the 100 videos, ensuring that the stimuli indeed depicted respective emotional expressions. Out of these 20 actors' emotional face videos, we selected 25 different emotional states (14 negative, 11 positive) for the Face Puzzle tasks with particular emphasis on emotions with high communicative valence (Hepach et al., 2011).

### Tasks

#### Face puzzle—implicit and explicit tasks

Face Puzzle consists of two independently applicable tasks for the assessment of implicit and explicit emotion recognition abilities from faces using dynamic and thus more naturalistic video-based stimulus material comprising basic as well as complex emotions. After stimulus production, task development and online implementation, we conducted a separate validation study using an additional sample of healthy individuals with an initial composition of the Face Puzzle implicit and explicit tasks (*N* = 29, mean age = 27.21, *SD* = 8.5, 13 male). According to the results, items were revised if necessary.

**Figure 1:**
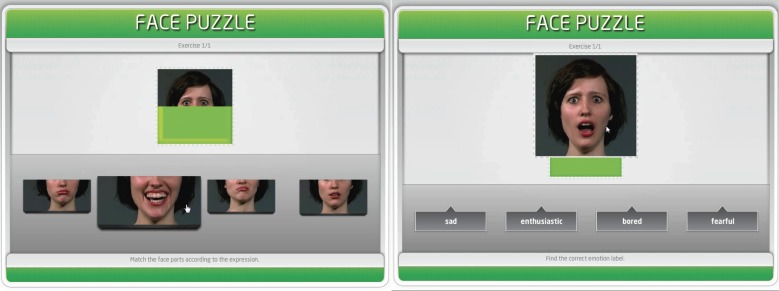
**Left:** implicit task. Participants have to find the according mouth video to the target eye-video. **Right**: explicit task. Participants have to explicitly label the target emotional expression.

Each task comprised 25 trials with one target emotional expression resulting in 25 different short video clips (mean length = 10.3 s) portrayed by in sum 15 professional actors [in each task: 7 male, varying age (20–50 years)] [see Figure 1 and supplementary video material (Movie 1 and Movie 2) showing example trials for the Face Puzzle implicit and explicit tasks]. Each actor portrayed between 1 and 3 emotions in different video clips (on average 1.8 emotions per task). In sum, five basic (angry, happy, disgusted, fearful, surprised) and 20 complex (interested, amused, aggrieved, troubled, jealous, enthusiastic, apologetic, disappointed, relieved, expectant, bored, compassionate, contemptuous, pardoning, embarrassed, wistful, furious, content, confident, doubtful) emotions (11 positive, 14 negative) were covered. We included a larger number of different emotional states than usually done in emotion recognition tasks to improve the tasks' sensitivity to real-life abilities/impairments instead of using only, e.g., the 6 basic emotions. No trial and thus no target emotion was repeated for each task. There was no feedback about whether an item was solved correctly or not in any of the tasks. To control for order effects, task order was counterbalanced across participants.

In the implicit task, face videos were divided into an upper (including the eye region; eye video) and a lower part (including the nose and the mouth region; mouth video). The target item represented an eye video displayed in the upper center of the screen. Four mouth videos of the same actor were displayed below the target item. The target eye video started playing automatically in loops, i.e., the video played repeatedly, until the participant completed the item by dropping a mouth video into the target field. Mouth videos were displayed as still images until participants directed the computer mouse on a video, thereby enlarging and starting to play the video. Participants then had to match the eye video with the respective mouth video according to the emotion presented in the facial mimic and place it underneath the eye video through a drag and drop function (see, Figure 1, left, Movie 1). No further information about the presented emotional states was given, so that participants had to process the depicted emotion in the face parts without explicitly being asked to identify or label a specific emotion. Actors were instructed to minimize head movement to prevent participants from matching parts solely due to general motion.

In the explicit task, the full target video (including both eyes and mouth) was presented in the upper center of the screen. Again, participants could enlarge the video for a more detailed inspection when directing the computer mouse above the item. As in the implicit task, the target video played automatically in loops, with the video automatically restarting until the item was completed. Participants were asked to choose the correct label for the presented emotion out of four emotion labels. The item was completed when the chosen label was placed into a target field below the target video through a drag and drop motion with the computer mouse. Distractor labels consisted of (1) two emotions of the same valence, one with similar valence and arousal levels and one that differed more in arousal level but had the same valence as the target item, and (2) one emotion of the opposite valence (see, Figure 1, right, Movie 2; for an example).

There was no time limit to respond to either task. Participants were asked to perform as fast and as accurately as possible. Both tasks were independent web-based applications that were accessible through a password-protected website. Completing each task took ~15–20 min. Tasks were designed and programmed in cooperation with a digital agency (gosub communications GmbH, www.gosub.de). The tasks could be accessed on a public webserver through any browser with a Flash Player Plugin installed. Both the implicit and explicit tasks began with a few introduction slides. Intuitive mouse interactions were used to avoid user distraction; throughout the entire application, participants navigated through introduction screens and solved the individual items only using the mouse.

### Procedure and measures

The participants completed both tasks online through the project's website in testing rooms of the Freie Universität Berlin, Germany under the supervision of trained experimenters. Performance measures comprised accuracy (percentage of correct answers) and reaction times in choosing the correct lower face part in the implicit task and the correct label in the explicit task, respectively. To account for the absence of a time limit to complete trials in both tasks, we calculated an individual composite measure for each participant and for each task, representing an ‘accuracy-adjusted response time’. The composite measure was calculated by dividing response times for correct items by the fraction of trials answered correctly (accuracy). Such a combinational measure of reaction time and number of correct responses is considered to account for compensatory strategies, e.g., speed-accuracy trade-off (e.g., Sucksmith et al., 2013) thereby representing a more sensitive measure of task performance.

The implicit task additionally recorded how often mouth videos were played and enlarged per trial (“playsum”), thereby also providing a measure for conscientious task execution in addition to the debriefing of participants after testing sessions.

To (1) investigate the new tasks' construct validity and (2) further differentiate between implicit and explicit emotion recognition processes, we additionally administered a standard explicit measure of emotion and mental state recognition from faces: the “Reading the Mind in the Eyes Test” (RMET, Baron-Cohen et al., 2001a). The RMET is a performance-based measure that requires participants to label emotional and mental states based on photographs of eye regions. To avoid possible ceiling effects in the control group, we additionally computed the subscale “*difficult*,” introduced by Domes et al. (2007), in addition to the RMET total score. The RMET aims to infer and explicitly label affective states, similar to the explicit Face Puzzle task. For further external validation of the Face Puzzle tasks and the potential dissociation of implicit vs. explicit socio-cognitive functioning, we additionally applied the “externally-oriented thinking” (EOT) subscale of the Toronto Alexithymia Scale (TAS-26, Kupfer et al., 2001, German version). The EOT-TAS scale measures the tendency to focus attention internally as opposed to externally, thus representing a measure of an implicit thinking style.

### Statistical analyses

Performance scores were first analyzed with repeated measures analysis of variance (ANOVA) to investigate potential main and interaction effects. Respective factors are specified in the results section. *Post-hoc t*-tests included independent samples *t*-tests. Reported *p*-values were adjusted for inhomogeneity if equal variances could not be assumed between groups and paired-samples *t*-tests within groups. Correlations between two measures were calculated based on Pearson's *r* correlation coefficients, whereas differences between correlations were calculated according to Fisher's *r*–*z* transformation. Reports of correlation include significance values (*p*), *z* statistics for Fisher's, as well as *t* and *r* statistics for Pearson's. All statistical tests used a significance threshold of *p* < 0.05 and were 2-tailed, if not specified otherwise.

## Results

### Sample information

Groups were matched with respect to age [*NT* = 30.29, *ASD* = 30.44, *t*_(46)_ = −0.92, *p* > 0.36], gender (NT and ASD group: 15 males, 9 females), and intelligence levels [vocabulary IQ test: *NT* = 106.21, *ASD* = 108.04, *t*_(46)_ = −0.53, *p* > 0.59; strategic IQ test: *NT* = 119.58, *ASD* = 120.54, *t*_(46)_ = −0.33, *p* > 0.73]. We additionally applied the Autism Spectrum Quotient (AQ, Baron-Cohen et al., 2001b) in both groups to control for clinically relevant levels of autistic traits in the NT group. As expected, the groups differed significantly in AQ scores [*NT* = 14.38, *ASD* = 37.37; *t*_(46)_ = 12.16, *p* < 0.001]. None of the controls scored above the cut-off score of 32; in fact, the highest score was 24, indicating a very low level of autistic traits in the NT group. Information on diagnostic scores of the ASD group is presented in Table 1. Please note that those ASD participants with no ADOS scores were diagnosed based on the ADI-R and *vice versa*. Thus, each ASD participants' diagnosis according to DSM criteria was confirmed with at least one of the two gold standard measures for diagnosing ASD.

**Table 1:** **Diagnostic scores for the ASD group (*n*, mean, minimum, maximum, *SE*, *SD*)**.

**Measure**	***n***	**mean**	**minimum**	**maximum**	***SE***	***SD***
ADOS	21	10.71	7	15	0.76	3.47
ADI-R	15	28.73	9	61	4.36	16.87
ASDI	21	42.29	34	56	1.09	4.99

Abbreviations: Autism Diagnostic Observation Schedule, ADOS; Autism Diagnostic Interview-Revised, ADI-R; Asperger disorder and High Functioning Autism Diagnostic Interview, ASDI; sample size, n; Standard Error of the Mean, SE; Standard Deviation, SD.

### Reliability analyses

#### Item analyses

We assessed the tasks' internal consistency by calculating Cronbach's alpha. Item difficulty was defined as the percentage of correct answers over all subjects divided by the total number of subjects (mean correct responses, see Wood, 1960). Both tasks demonstrated satisfactory reliability (*N* = 48, implicit task: Cronbach's alpha = 0.81, mean item difficulty = 0.69, range = 0.25–94; explicit task: Cronbach's alpha = 0.81, mean item difficulty = 0.74, range = 0.54–0.94).

The implicit task additionally offered the opportunity to control whether the participants performed the task in accordance with the instruction. We measured how often participants chose to play each of the four mouth videos for >1 s, thereby sufficiently inspecting presented emotional information (“playsum”). Among all the participants, the mean playsum was >4 (mean = 4.7, *SD* = 1.1), indicating that participants sufficiently inspected the emotional information provided in the video parts. Over all 25 trials, participants in the ASD group showed a trend toward a greater number of average playsum per trial [*NT* = 4.38, *ASD* = 4.97, *t*_(46)_ = −1.9, *p* = 0.06]. In other words, the ASD group tended to play the mouth videos more often than the NT group, over all trials.

In addition to the average playsum over all trials, we calculated playsum for correct and incorrect trials separately per participant and group. A 2 × 2 ANOVA with the within subject factor CORRECT (playsum for correct vs. incorrect trials) and the between subjects factor GROUP (ASD vs. NT) showed a significant main effect of CORRECT [*F*_(1, 46)_ = 21.01, *p* < 0.001, η^2^ = 0.3] but no main effect of GROUP or an interaction of the two factors (*p* > 0.14). Over both groups, participants played the mouth videos more often for incorrectly as compared to correctly solved items.

### Tasks' sensitivity to atypical emotion recognition

To test the new tasks' sensitivity to atypical emotion recognition we analyzed performance measures between and within groups.

#### Accuracy

**Figure 2:**
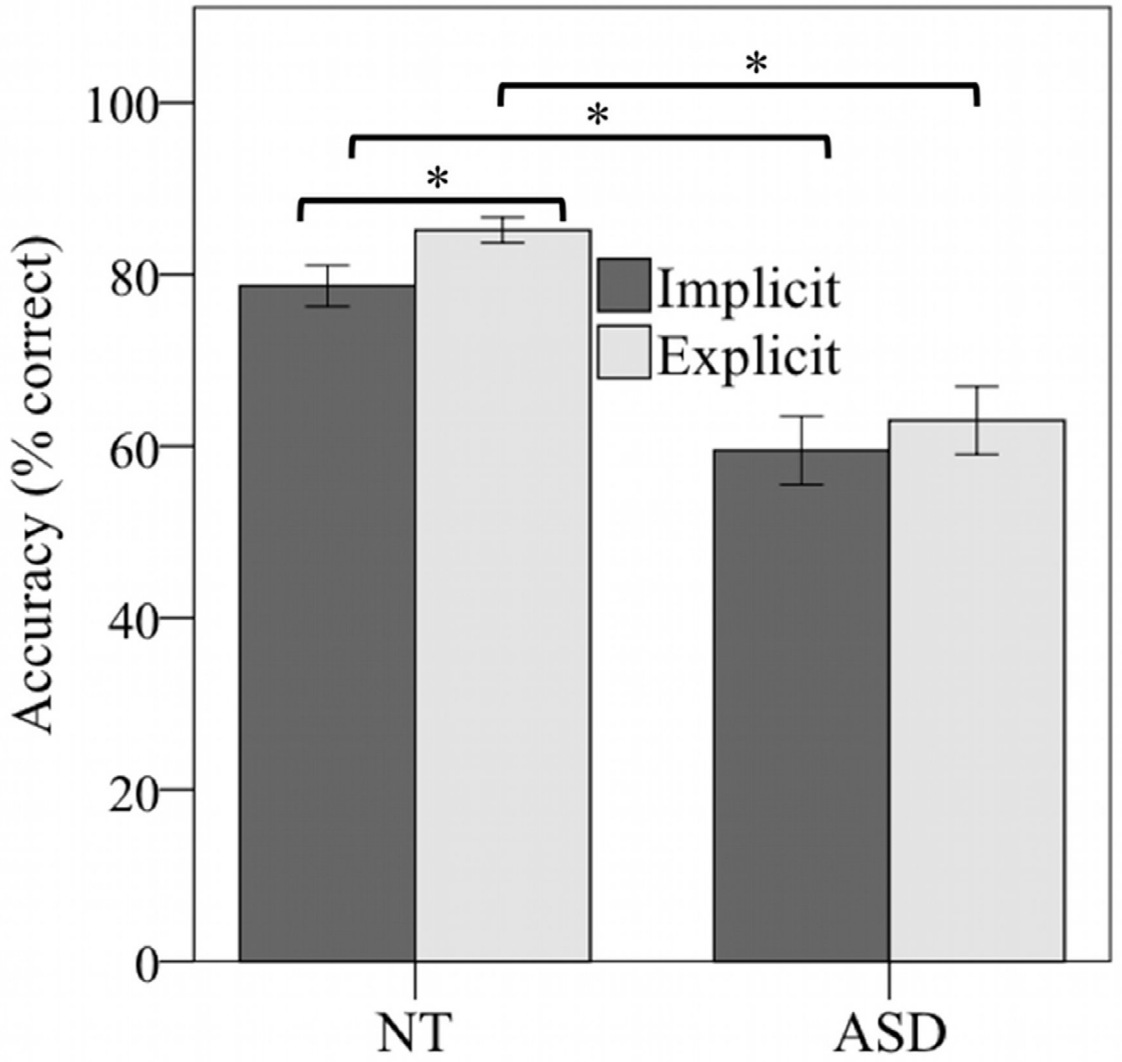
**Accuracy scores (percentage of correct responses) by TASK (implicit vs. explicit) and GROUP (ASD vs. NT).** Whereas there was no significant difference between implicit and explicit task accuracy in the ASD group, typically developed participants' accuracy was significantly increased in the explicit task. The NT group showed increased accuracy in both tasks as compared to the ASD group. ^*^p < 0.05.

Among all participants, the accuracy scores were considerably above chance level (i.e., 25%) and greater in the explicit than the implicit task (% correct responses: implicit = 69.08, explicit = 74.17). A 2 × 2 repeated measures ANOVA with the within-subjects factor TASK (implicit vs. explicit) and the between-subjects factor GROUP (NT vs. ASD) yielded significant main effects of TASK [*F*_(1, 46)_ = 5.08, *p* = 0.029, η^2^ = 0.1] and GROUP [*F*_(1, 46)_ = 29.35, *p* < 0.001, η^2^ = 0.34]. There was no interaction between TASK and GROUP on the mean accuracy scores (*p* > 0.5, η^2^ = 0.1). Despite the absence of this interaction, we nevertheless analysed group-specific data to further inform atypical and typical emotion recognition in the implicit and explicit tasks in an exploratory fashion. Across all items, the groups' accuracies differed significantly for each task [implicit: *NT* = 78.67, *ASD* = 59.5, *t*_(46)_ = 4.13, *p* < 0.001; explicit: *NT* = 85.17, *ASD* = 63.17, *t*_(46)_ = 5.26, *p* < 0.001] (see, Figure 2). Within the NT group, accuracies between tasks differed significantly with increased performance in the explicit task [implicit = 78.67, explicit = 85.17, *t*_(23)_ = −2.61, *p* < 0.016], while there was no such effect in the ASD group [implicit = 59.5, explicit = 63.0, *t*_(23)_ = −0.92, *p* = 0.37].

***Valence effects on task accuracy***. We conducted additional analyses on accuracy scores with regard to valence direction (positive vs. negative) and valence strength in both Face Puzzle tasks. Valence strength of emotion words (not videos) were taken from Hepach et al. (2011), which provided valence and arousal ratings of 40 emotional words, including the 25 used as target emotional states in the new Face Puzzle tasks (see methods section). We then built new accuracy scores for low/high valence strength within the positive and negative words and performed a 2 × 2 × 2 ANOVA with the within subject factors VALENCE (positive vs. negative) and STRENGTH (low vs. high) and the between subject factor GROUP (ASD vs. NT). The ANOVA yielded a significant main effect of STRENGTH [*F*_(1, 46)_ = 36.6, *p* < 0.001, η^2^ = 0.44] and a significant interaction of strength and valence [*F*_(1, 46)_ = 5.43, *p* = 0.024, η^2^ = 0.11]. Over all participants, emotions with lower valence strength were more often correctly identified than those with higher valence strength (positive vs. negative) [*t*_(1, 47)_ = −6.08, *p* < 0.001], whereby the magnitude of this effect was greater for positive [*t*_(1, 47)_ = −4.89, *p* < 0.001] than for negative emotions [*t*_(1, 47)_ = −2.19, *p* = 0.033].

***Analyses of error types***. We performed analyses with regard to the types of errors for both Face Puzzle tasks. According to the three types of incorrect response options per trial and task [error type 1 (ET1): same valence and similar arousal as compared to the target item's emotion, error type 2 (ET2): same valence and more distant arousal, error type 3 (ET3): different valence], we defined three new variables reflecting the percentage of specific error types over the total number of errors over all trials per task and group. We then performed a 2 × 3 × 2 ANOVA with the within subject factors TASK (implicit vs. explicit) and ERRORTYPE (ET1 vs. ET2 vs. ET3), between the subject factor GROUP (NT vs. ASD). This ANOVA revealed a significant main effect of ERRORTYPE [*F*_(1, 46)_ = 5.19, *p* = 0.007, η^2^ = 0.1] and a significant interaction of TASK and ERRORTYPE [*F*_(1, 46)_ = 12.68, *p* < 0.001, η^2^ = 0.22], but no interaction with the factor GROUP.

*Post-hoc* paired-samples-*t*-tests showed that over both tasks ET3 was significantly less often selected than ET2 over all participants, [*t*_(1, 47)_ = 2.59, *p* = 0.013] and ET1 [*t*_(1, 47)_ = 3.02, *p* = 0.004]. ET1 did not differ between the Face Puzzle implicit and explicit tasks [*t*_(1, 47)_ = 0.013, *p* > 0.9]. In contrast, ET2 and ET3 were significantly different between tasks. Whereas ET2 was significantly more often selected in the Face Puzzle implicit as compared to explicit task [*t*_(1, 47)_ = −3.62, *p* = 0.001], ET3 appeared significantly more often in Face Puzzle explicit as compared to implicit [*t*_(1, 47)_ = 4.76, *p* < 0.001].

#### Reaction times

Mean reaction times for correct responses were calculated for each participant in both tasks and are referred to as RTs. Trials with incorrect responses were excluded from further analyses. We expected the RTs to differ between the implicit and explicit tasks, given that the tasks differed systematically in their answering format. In the implicit task, RTs represent the time between the start of the trial and the drop of a mouth video into the target field, including the time to inspect the target eye video and the mouth video options. In contrast, in the explicit task, participants were only presented with one face video and four emotion words. As mentioned previously, there were no time limits to respond.

As expected, the RTs differed systematically between tasks, and all participants were faster in responding correctly for the explicit as compared to the implicit task [NT: implicit = 32.12 s, explicit = 10.92 s, *t*_(23)_ = 11.38, *p* < 0.001; ASD: implicit = 44.77 s, explicit = 16.27 s, *t*_(23)_ = 7.46, *p* < 0.001]. A 2 × 2 ANOVA with the within-subjects factor TASK (implicit vs. explicit) and the between-subjects factor GROUP (NT vs. ASD) additionally replicated the significant main effect of TASK [*F*_(1, 46)_ = 137.53, *p* < 0.001, η^2^ = 0.75] and GROUP [*F*_(1, 46)_ = 8.62, *p* = 0.005, η^2^ = 0.16], as well as showing a trend toward an interaction of both factors [*F*_(1, 46)_ = 2.69, *p* = 0.1, η^2^ = 0.06]. In analogy to the accuracy data analyses, we performed further *post-hoc* independent samples *t*-tests despite the interaction did not reach significance, to inform reaction times during atypical and typical emotion recognition in an exploratory fashion. The NT group was generally faster in responding than the ASD group in both the implicit as well as the explicit tasks [implicit: *NT* = 32.13 s, *ASD* = 44.77 s, *t*_(46)_ = −2.48, *p* = 0.019; explicit: *NT* = 10.92 s, *ASD* = 16.27 s, *t*_(46)_ = −3.24, *p* = 0.002].

***Analyses of individual reaction time variance***. To test for possible individual variance differences in RTs between groups, we calculated individual inter-trial variability for each task separately, as measured with standard deviation of RTs. There were no significant group differences in RT variance for the Face Puzzle implicit task [*NT* = 5.4, *ASD* = 6.95, *t*_(1, 46)_ = −1.38, *p* > 0.178, equal variance not assumed]. For the Face Puzzle explicit task there was only a trend toward a group difference [*NT* = 13.69, *ASD* = 17.87, *t*_(1, 46)_ = −1.85, *p* > 0.073, equal variance not assumed].

#### Composite measure of reaction times and accuracy

**Figure 3:**
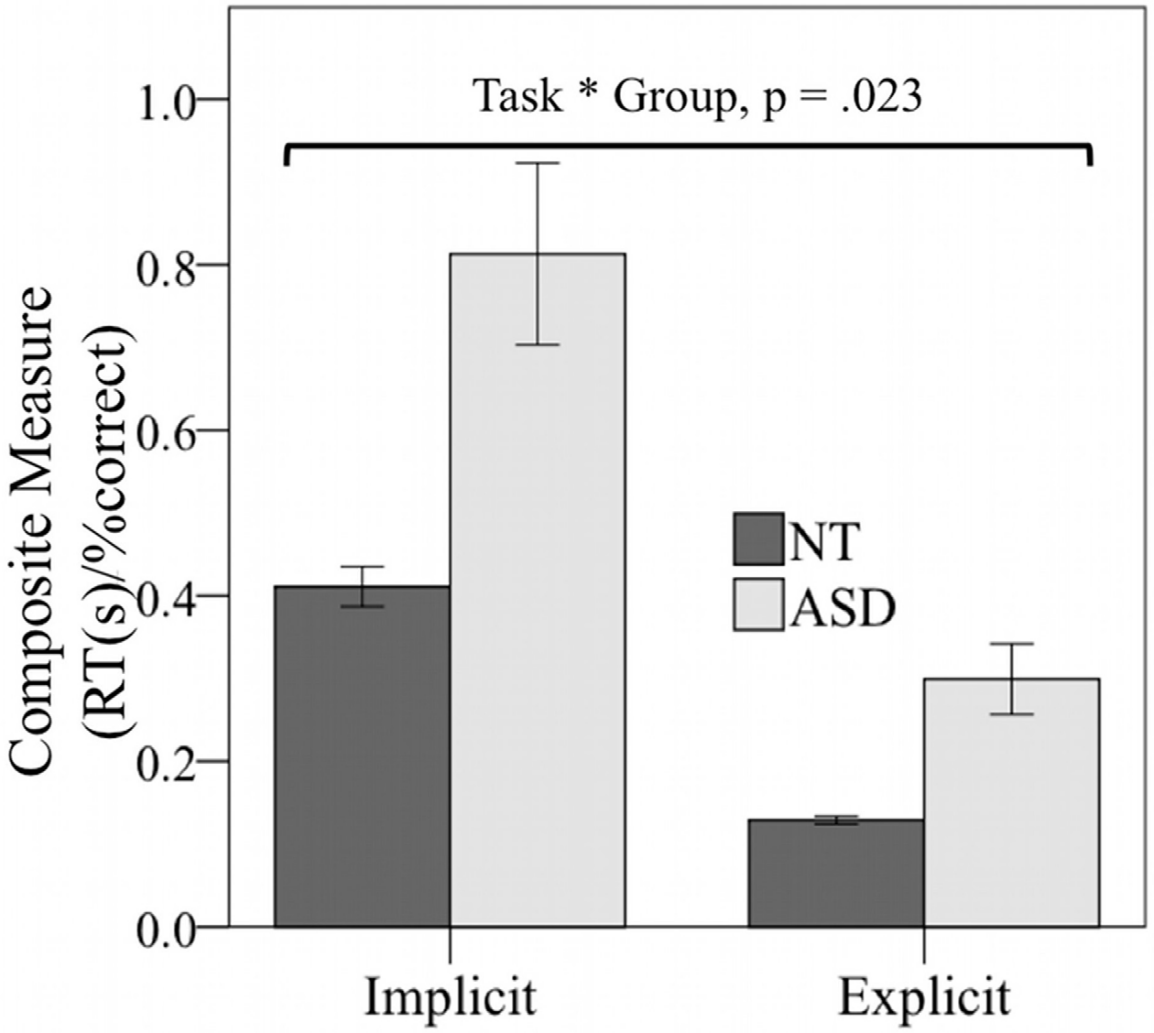
**Significant TASK (implicit vs. Explicit) by GROUP (ASD vs. NT) interaction of the composite measure [RT (seconds)/% correct responses] (*p* = 0.023).** The group difference was greater for the implicit as compared to the explicit task, indicating increased implicit processing impairments in ASD. Abbreviations: Reaction Times, RT; seconds, s.

As outlined in more detail in the methods section, we calculated a composite measure (reaction times divided by accuracy) to account for the absence of a time limit to complete trials in both tasks. Overall, participants yielded lower values in the implicit as compared to the explicit task (see, Figure 3). A 2 × 2 repeated-measures ANOVA [TASK (implicit vs. explicit) × GROUP (NT vs. ASD)] showed a main effect of TASK over both groups [*F*_(1, 46)_ = 65.17, *p* < 0.001, η^2^ = 0.59] and a main effect of the factor GROUP [*F*_(1, 46)_ = 17.1, *p* < 0.001, η^2^ = 0.27]. The composite measure was additionally mediated by a significant interaction with the factor GROUP [*F*_(1, 46)_ = 5.5, *p* = 0.023, η^2^ = 0.11]. As outlined in Figure 3, the magnitude of the group difference was greater for the implicit than the explicit task. For both tasks, the NT group showed significantly lower values than the ASD group [implicit: *NT* = 0.41, *ASD* = 0.81, *t*_(46)_ = −3.5, *p* = 0.001; explicit: *NT* = 0.13, *ASD* = 0.29, *t*_(46)_ = −3.9, *p* < 0.001], indicating improved performance in general.

### Relationship between implicit and explicit performance and diagnostic scores in ASD

**Figure 4:**
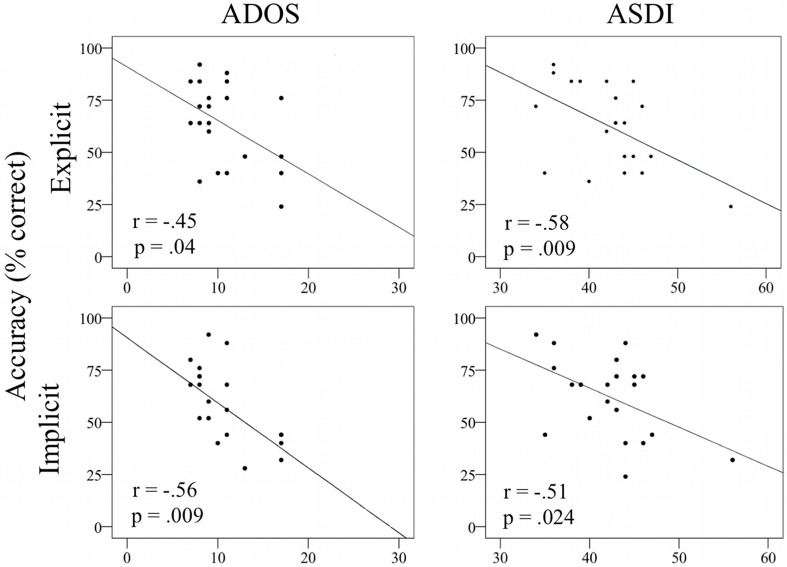
**Scatter plots displaying the correlation between diagnostic scores and accuracy (% correct responses) in the Face Puzzle implicit and explicit task for the ASD group. Upper:** Face Puzzle explicit task, **lower**: Face Puzzle implicit task, **left**: ADOS, **Right**: ASDI. Abbreviations: Autism Diagnostic Observation Schedule, ADOS; Asperger Syndrome and High Functioning Autism Diagnostic Interview, ASDI.

The ASD participants' accuracy scores were significantly correlated with autism symptomatology, as measured by the ADOS [implicit: *r*_(19)_ = −0.55, *t* = −2.87, *p* = 0.009; explicit: *r*_(19)_ = −0.45, *t* = −2.2, *p* = 0.04] and the ASDI [implicit: *r*_(19)_ = −0.49, *t* = −2.45, *p* = 0.025; explicit: *r*_(19)_ = −0.52, *t* = −2.65, *p* = 0.016]. The more severely affected individuals scored lower on both tasks, indicating a link between symptom severity and task performance and revealing the sensitivity of the new tasks to atypical emotion recognition performance (see Figure 4).

### Correlations with external measures

To assess whether accuracy in the implicit and explicit tasks differed in relation to external implicit and explicit socio-cognitive measures, we performed further correlation analyses with the externally orienting thinking scale of the TAS and the RMET, respectively. The EOT-TAS scale measures the extent to which individuals orient their thinking internally without external prompts, thus providing a measure for the individual magnitude of implicit processing when processing emotions. In contrast, the RMET asks participants to label emotional stimuli with provided labels, representing a classic explicit labeling task in emotion and mental state recognition.

Analyses for the RMET scores were performed separately for each group, given that the groups differed significantly. Because the groups did not differ in the EOT-TAS subscale, we performed the respective correlations across all participants (see Table 2).

**Table 2:** **Group means, *SD*, range and group differences in accuracy and mean scores for the external socio-cognitive measures**.

	**RMET^+^**	**RMET^+^ diff**	**EOT (TAS)**
	**Accuracy**		**Mean score**
***NT***			
*M*	73.46	69.06	13.58
*SD*	7.68	10.5	3.2
Range	58–83	44–83	8–19
***ASD***			
*M*	63.03	56.9	15.38
*SD*	14.67	13.97	4.44
Range	26–83	33–78	8–24
*p-value*	0.004^*^	0.002^*^	0.12

Abbreviations: Reading the Mind in the Eyes Test difficult item scale, RMET diff; Externally-Oriented Thinking style scale, EOT; Toronto-Alexithymia Scale, TAS; Mean, M; Standard Deviation, SD. p-values: two-tailed significance-value for independent samples t-tests between ASD and NT participants;

* < 0.05;

+ sample sizes differed for each group: RMET^+^: NT: 24, ASD: 19.

#### Explicit processes

The NT group's accuracy in the explicit Face Puzzle task correlated significantly with performance on the explicit external measure *difficult RMET* items [Pearson's *r*_(22)_ = 0.44, *t* = 0.2.3, *p* = 0.033], whereas there were no significant correlations between accuracy in the implicit task and the RMET scores (all *p* > 0.15). The correlation coefficients for the implicit and explicit accuracy with the RMET scores did not differ significantly (*z* > 1.1, *p* > 0.2) (see Table 3).

**Table 3:** **Correlational analyses between Face Puzzle Task and external socio-cognitive measures**.

	**EOT (TAS)**	**RMTE diff^+^**	
	***NT and ASD***	***NT***	***ASD***
FP-I	*r* = −0.28	*r* = 0.14	*r* = 0.43
	*p* = 0.051	*p* > 0.51	*p* = 0.068^(*)^
FP-E	*r* = −0.23	*r* = 0.44	*r* = 0.51
	*p* = 0.11	*p* = 0.033^*^	*p* = 0.024^*^

Abbreviations: accuracy scores in the Face Puzzle implicit, FP-I and explicit task, FP-E; Externally-Oriented Thinking style scale, EOT; Toronto-Alexithymia Scale, TAS; r-values: Pearson's correlation coefficient; p-values: two-tailed significance-value for bivariate correlation,

(*) < 0.1;

* < 0.05;

+ sample sizes differed for each group: RMET^+^: NT: 24, ASD: 19.

In the ASD group, RMET scores correlated with accuracy in the explicit as well as the implicit task significantly or on trend level [RMET: implicit: *r*_(19)_ = 0.38, *t* = 0.17, *p* = 0.11, explicit: *r*_(19)_ = 0.55, *t* = 2.72, *p* = 0.015; *difficult RMET* items: implicit: *r*_(19)_ = 0.43, *t* = 1.96, *p* = 0.068, explicit: *r*_(19)_ = 0.51, *t* = 2.45, *p* = 0.024].

#### Implicit processes

Across all participants, the EOT-TAS scores' correlation with accuracy in the implicit task barely missed significance [*r*_(46)_ = −0.28, *t* = −1.98, *p* = 0.051]. In contrast, the EOT-TAS scores did not correlate significantly with accuracy in the explicit task (*p* > 0.1). These correlations did not differ significantly between the implicit and explicit scores (*z* = −0.024, *p* > 0.8).

### Dissociating implicit and explicit processes

We further conducted correlation analyses on the composite measure (accounting for both accuracy and RTs within and between groups) accuracy, and reaction times to assess potentially differential relations and to investigate dissociations between implicit and explicit emotion recognition processes.

**Figure 5:**
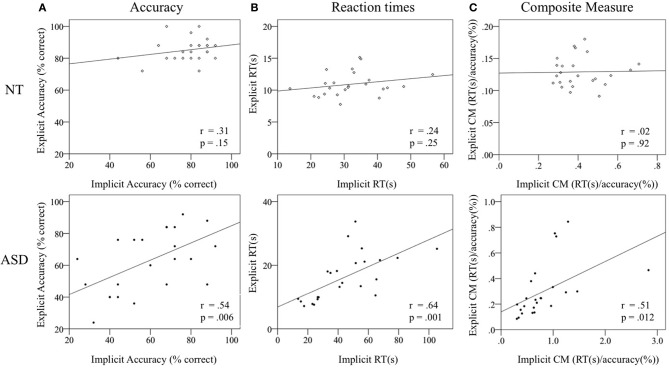
**Scatter plots displaying the correlations of accuracy (A), reaction times (B) and composite measure (C) scores per group between the Face Puzzle implicit and explicit task.** Upper: NT group, lower: ASD group. Please note that scatter plots are presented separately for each group with group specific scales according to the group range to inform within group correlations. Abbreviations: Composite Measure, CM; Reaction Times, RT; seconds, s.

In the NT group, the composite scores, RTs and accuracy scores for the implicit and explicit tasks were not correlated [composite measure: *r*_(22)_ = 0.022, *t* = 1.2 *p* = 0.92, accuracy *r*_(22)_ = 0.31, *t* = 0.7, *p* = 0.15, RTs: *r*_(22)_ = 0.24, *t* = 1.16, *p* = 0.25]. In contrast, performance correlated significantly between tasks in the ASD group [composite measure: *r*_(22)_ = 0.51, *t* = 2.78, *p* = 0.012, accuracy *r*_(22)_ = 0.54, *t* = 3.01, *p* = 0.006, RTs: *r*_(22)_ = 0.64, *t* = 3.9, *p* = 0.001]. Correlations between groups differed by marginal significance for the composite measure (*z* = −1.73, *p* = 0.08) and reaction times (*z* = −1.6, *p* = 0.09), indicating a different relationship between implicit and explicit emotion recognition performance in the NT as compared to the ASD group (see Figure 5 for group specific scatter plots of the composite measure correlation).

## Discussion

The current study aimed at developing a new tool for comparably assessing implicit and explicit aspects of facial emotion recognition with two new tasks: Face Puzzle implicit and explicit. Aiming at a more naturalistic assessment of social cognitive processes, we included basic as well as more complex emotions expressed by a number of different actors in life-like video stimuli. To test (1) the tasks' reliability and validity, as well as (2) their sensitivity to atypical social cognition, we applied the Face Puzzle implicit and Face Puzzle explicit tasks to a healthy sample as well as to age-, gender- and intelligence-matched adults with ASD. In sum, our results identified the Face Puzzle tasks as reliable (both: alpha > 0.8), externally valid, and sensitive to atypical social cognition.

### An approach toward measuring implicit and explicit aspects of facial emotion recognition

The development of human cognition, including social cognition, is comprised of the specification of basic spontaneous reactions into explicit representations and concepts, which can be externally triggered and applied within respective contexts. Given the importance of identifying implicit and explicit processing aspects and associated performance within social cognition (Zaki and Ochsner, 2009, 2011), a respective theoretical dissociation model must be empirically tested and validated to inform particular impairments in psychiatric conditions or atypical development. With the two new tasks introduced in the current study we pursued one possible approach toward a performance-based comparison of implicit and explicit emotion recognition processes.

To further investigate the external validity of the new tasks, we correlated performance with established socio-cognitive measures. The performance of typically developed individuals in the explicit task correlated with accuracy in the RMET task, which also provides explicit prompts to identify facial emotions, while there was no correlation with implicit task performance in healthy controls. Performance in the implicit task correlated marginally significantly with the tendency to orient thinking strategies to internal as opposed to external cues, as measured by the EOT-TAS subscale, but no such correlation was found with performance in the explicit task in all participants. Despite the lack of significant differences in respective correlations with implicit/explicit measures, these results support the tasks' external validity.

### Implicit and explicit processing impairments in ASD

The ASD group showed reduced performance in both the Face Puzzle implicit and explicit tasks. This reflects the (1) overall decreased emotion recognition and (2) specific impairments in implicit as well as explicit processes within facial emotion recognition. Potential reasons for reduced performance could lie in a lack of social motivation (e.g., Schultz et al., 2003; Schultz, 2005) or an aversiveness of direct eye contact (e.g., Hutt and Ounsted, 1966; Kliemann et al., 2010), which would lead to less exposure and thus less expertise in encoding emotions from faces. In particular, we found that groups differed to a greater magnitude in the implicit as compared to the explicit Face Puzzle task in the composite measure, as indicated in the interaction with the factor group. Previous studies' findings showed severe impairments in implicit processing of social information in the absence of explicit cues in high-functioning ASD (Kaland et al., 2011; Senju, 2012). In addition, Volkmar and colleagues (see e.g., Volkmar et al., 2004) have proposed that social impairments in ASD are specifically evident in unstructured settings. Our results of greater between group differences in the Face Puzzle implicit task as assessed by the composite measure and reaction times thus underlines a more pronounced impairment in implicit than explicit processing of facial emotion information in ASD, as compared to controls. Noteworthy, however, we did not find a greater magnitude of differences between groups in accuracy alone. We expect that the absence of a time limit to complete trials in both tasks contributed to this. A modulated version of the tasks with a restricted time limit to respond (based on the control group's reaction time data, for instance) could be used to test this hypothesis.

### Relation of implicit and explicit processes in typical and atypical development

The two new Face Puzzle tasks allowed us to compare performance in implicit and explicit emotion processing intra- and inter-individually. The particular task relations were informative with respect to the question of whether the proposed dual-process models in other cognitive domains or social cognitive abilities [e.g., ToM, (Apperly and Butterfill, 2009; Low and Perner, 2012)] can be applied to emotion recognition from faces. The current results suggest a behavioral dissociation of implicit and explicit aspects of emotion recognition in typical development as the NT group's performance in the explicit Face Puzzle task differed significantly from that in the implicit Face Puzzle task. Furthermore, performance in terms of accuracy, reaction times, and the composite measure in both tasks was not intercorrelated for the NT group. We do not interpret this as indication that implicit and explicit processes are independent *per se* in typically developed individuals. Instead, the data suggests that the respective processes can be independently assessed to a certain extent with the new Face Puzzle tasks, implying the possibility of autonomous operation given specific task demands. In contrast, the ASD group's performance did not differ between tasks and was correlated significantly, indicating a lack of behavioral dissociation of implicit and explicit aspects of emotion recognition in atypical development. Because the new tasks did not restrict participants in their decision time, investigating performance with a composite measure and combining both accuracy and reaction times provided a more comprehensive approach (Sucksmith et al., 2013). Correlations between the composite scores differed between the groups on a trend level, again suggesting a differential relationship between implicit and explicit aspects of emotion recognition in typical and atypical development.

For general and social cognition, implicit processing has been suggested as a developmental precursor to explicit processing (Karmiloff-Smith, 1992; Dienes and Perner, 1999). Our data suggest that this notion also applies to facial emotion recognition abilities, as the correlation of both implicit and explicit task performance with the explicit RMET scores in the patient group suggested an aberrant dissociation of implicit and explicit emotion recognition in ASD. Abnormal implicit processing of social stimuli during development would likely result in (1) impaired explicit processing and (2) the lack of functionally dissociable implicit and explicit systems, as suggested by the current study's group-specific results. Given the nature of the study's cross-sectional design in adults and our particular conceptualization of implicit processing, however, this interpretation is tentative and should be further tested in longitudinal designs with different age groups and based upon further operationalization of implicit and explicit emotion recognition processes.

### Implications for future research and interventions

Previous studies have mostly assessed aspects of implicit (facial) emotion processing indirectly, using techniques such as backward-masking (see, e.g., Pessoa, 2005) or age and gender differentiation tasks (see, e.g., Habel et al., 2007). The indirect assessment of implicit emotion processing does not, however, allow for a direct comparison to explicit processes. Here, we attempted to measure implicit and explicit aspects of emotion recognition processes based on performance in comparable and easily applicable tasks. In contrast to conceptualizations in other fields, such as social or developmental psychology, in our study, “implicit” was operationalized more narrowly and did not involve consciously controlled processing of provided emotional labels. We consider this as one possible approach toward measuring implicit processes to make them comparable to explicit processes with the new Face Puzzle tasks. With regard to the literature on implicit processing of social information in general (e.g., Hudson et al., 2009; Hudson and Jellema, 2011) and related impairments in ASD (Jellema et al., 2009; Hudson et al., 2012; Senju, 2013), previous studies used mostly tasks that assessed unconscious and involuntary processing of social information. For example, a study by Jellema et al. (2009) found impairments in involuntarily interpreting social cues in ASD. In contrast, the current study involved less unconscious/more conscious processing of facial information, but crucially without explicitly providing verbal labels (e.g., find the happy mouth to the happy eyes), thus representing a theoretically different operationalisation. Future studies should carefully relate and compare abilities and related deficits in different aspects of implicit processing to systematically investigate the influence of conscious vs. unconscious processing of social information.

Quantifying the accuracy of implicit processing can help providing individual targets for interventions that aim to improve socio-cognitive functioning (Zaki et al., 2010; Zaki and Ochsner, 2011). Interventions to date have mostly emphasized explicit emotion recognition, such as by training individuals to assign verbal labels to presented emotional information (see, e.g., FEFA, Bölte et al., 2006; Golan et al., 2006). Using the example of ASD, the identification of greater impairments in processing implicit aspects within social cognitive functioning in general and facial emotion recognition in particular underlines the need to place a focus on respective processes in interventions. Future trainings could, for instance, focus on those implicit aspects of social cognition in everyday life by making them more explicit via detailed descriptions or categorizations to promote compensatory effects to support effective social functioning.

Despite the instruction to respond as fast as possible in both Face Puzzle tasks, the current task versions had no pre-defined time limit for participants to give a response. The current study thus provides the first reference points for mean and range of response times of typically developed individuals as well as samples that are impaired in emotion recognition. Based on these data, future studies could explore task accuracies with a pre-defined response window and investigate (1) the effect of time constraints on participants behavior, as well as (2) influence of confidence on decision time and accuracy (for instance, by collecting confidence ratings of decisions after each trial).

We did not find group interactions regarding valence effects of emotional stimuli. Importantly, information on valence strength were behavioral ratings of emotional words from Hepach et al. (2011) and thus only indirectly related to the actual strength and/or intensity of the emotional expressions in the videos. It may be thus informative to acquire valence strength ratings for the Face Tasks' actual videos and relate these ratings to performance of healthy samples and those impaired in emotion recognition.

Further, there were no group differences or interactions in types of errors in both Face Puzzle tasks. Noteworthy, in the Face Puzzle explicit task the likelihood of choosing the label reflecting error type 3 (different valence) may be reduced as compared to the Face Puzzle implicit task. In the Face Puzzle implicit task, valence difference could have been less obvious and explicit in the videos than in the labels. In fact, ANOVA regarding error types showed this effect with a higher number of error type 3 in the Face Puzzle implicit than in the explicit task. Consequently, between-task comparisons of error type were influenced by task specific presentation of distractor options and can thus not be easily compared. Error type analyses between tasks in future studies should thus take this task interaction into account.

### Limitations

Here, we proposed one possible approach to operationalize implicit processing by asking participants to complete a puzzle of facial expressions. However, future research is needed to extend and further test this conceptualization and to explore other possible ways to allow implicit and explicit processing to be compared.

It is important to note that the external validation of new tasks is dependent on correlations with established measures that assess the psychological construct of interest as well as possibly confounding processes. While we have included tasks in our study design that reflect implicit and explicit emotion processing strategies, we have not considered other cognitive functions that might contribute to performance in our implicit Face Puzzle task. For example, the perception of motion and the ability to holistically process gestalt, both of which have been shown to be compromised in autism (see, e.g., Gauthier et al., 2009), may be present and could have contributed to the observed effects. It remains an unresolved question whether and to which extend those and other potentially interacting constructs/confounds contribute to implicit social processing in general and to more implicit aspects of facial emotion recognition in particular. This should be carefully explored in future studies.

In addition, the EOT scale of the TAS is a rather distant external measure of “implicit facial emotion processing” and more closely related to general autonomous-mechanistic (Marty and De M'uzan, 1978) and thus implicitly oriented thinking styles. Relations between performance on the Face Puzzle implicit task and other measures of implicit social information processing, e.g., by analysis of gaze or other tasks that approximate less conscious aspects of implicit social processing (such as Jellema et al., 2009) should be tested and validated.

It is further noteworthy that the explicit task might have been easier (in terms of task difficulty) than the implicit task, especially for the NT group. By acquiring more data with the new Face Puzzle tasks, thereby increasing the number of observations in healthy participants (e.g., with varying verbal IQ levels and thus potentially creating a greater variance in NT data), this hypothesis should be clearly tested.

As outlined above, implications for the development of implicit and explicit processes in atypical social cognition, with the example of ASD, are only of an indirect nature given the adult samples and the cross-sectional design of the current study. We encourage future studies to directly test the proposed aberrant developmental trajectory.

## Conclusion

In sum, the current study introduces Face Puzzle, which consists of two new computer-based tasks for measuring implicit and explicit aspects of facial emotion recognition performance with more naturalistic video stimulus material and basic as well as complex emotions. Item analyses, correlations with established external socio-cognitive measures and performance differences between typically (healthy) and atypically (ASD) developed individuals suggest reliability, validity and sensitivity of the Face Puzzle implicit and explicit tasks. Furthermore, the magnitude of performance differences between the groups indicates a particular emphasis on implicit processing deficits in ASD along with first hints toward group specific relations of explicit and implicit aspects of facial emotion recognition processes.

### Conflict of interest statement

The authors declare that the research was conducted in the absence of any commercial or financial relationships that could be construed as a potential conflict of interest.
